# Chronic hepatitis B virus infection increases the risk of upper urinary calculi

**DOI:** 10.1186/s12894-022-01038-z

**Published:** 2022-06-06

**Authors:** Bingbing Hou, Changming Lin, Zongyao Hao

**Affiliations:** 1 Department of Urology, The First Affiliated Hospital of Anhui Medical University, No. 218 Jixi Road, Hefei, 230032 China; 2 Institute of Urology, Anhui Medical University, Hefei, China; 3 Anhui Province Key Laboratory of Genitourinary Diseases, Anhui Medical University, Hefei, China; 4 Department of Urology, The Fourth Affiliated Hospital of Anhui Medical University, Hefei, China

**Keywords:** Urinary calculi, Hepatitis B virus, Risk factors, Urine pH, Urolithiasis

## Abstract

**Background:**

Although hepatitis B virus (HBV) is a recognized risk factor for renal diseases, little is known about HBV infection in individuals with upper urinary calculi (UUC). We investigated the relationship between chronic HBV infection and UUC.

**Methods:**

We retrospectively analysed data from 1399 patients who were discharged from the Department of Urology (2017–2018). The diagnosis of UUC was determined using urinary tract ultrasonography or computed tomography, and HBV infection was evaluated by a positive hepatitis B surface antigen (HBsAg) test. Data on patients with and without UUC and HBsAg-positive and HBsAg-negative patients were compared by univariate and multivariate analyses.

**Results:**

Data on chronic HBV infection and UUC were available for 1062 patients, including 514 who presented with UUC and 548 who did not. Overall, 5.8% of total patients, 8.0% of UUC patients and 3.8% of non-UUC patients had chronic HBV infection. UUC patients (41/514) had a significantly higher prevalence of HBsAg positivity (OR 2.175; 95% CI 1.267–3.734; *P* = 0.004) than non-UUC patients (21/548). After stratifying by sex, the relative odds of HBsAg positivity were statistically significant in men (OR 2.156; 95% CI 1.162–4.003; *P* = 0.015) but not in women (OR 2.947; 95% CI 0.816–10.643; *P* = 0.099). The incidence of urinary pH > 6 and staghorn stones was significantly higher in HBsAg-positive UUC patients than in HBsAg-negative UUC patients.

**Conclusion:**

This is the first study to demonstrate that chronic HBV infection is strongly associated with UUC, at least in men. The urinary pH > 6 and staghorn stones were more common in UUC patients with chronic HBV infection.

**Supplementary Information:**

The online version contains supplementary material available at 10.1186/s12894-022-01038-z.

## Background

Globally, the prevalence and recurrence rates of kidney stone disease are increasing [1, 2]. It affects all ages, both sexes and all races; however, it occurs more commonly among males than females aged 40–60 [3, 4]. Approximately 10–12% of the population will be affected by kidney stones at some stage of their lives^.^ [2, 5] Therefore, the loss of working time and treatment involves considerable costs that seriously affect people’s quality of life and increase the national economic burden [6].

In addition to kidney stones, hepatitis B virus (HBV) infection is also a serious global public health problem. The WHO estimates indicate that approximately 240 million people worldwide are infected with HBV. HBV infection is the main cause of liver disease and has many extrahepatic manifestations [7]. It is well-known that HBV infection is also one of the recognized pathogenic factors for the formation of chronic kidney diseases [8, 9]; however, there are no studies on the prevalence of HBV infection in individuals with kidney stones.

We retrospectively analysed discharged urological patients. Urinary tract ultrasonography or computer tomography and hepatitis B surface antigen (HBsAg) testing were both performed. The primary outcome was to investigate the prevalence of HBV infection in patients with upper urinary calculi (UUC).

## Methods

### Study population and design

We retrospectively analysed data from 1399 patients who were discharged from the Department of Urology between January 2017 and December 2018 at the Affiliated Hospital of Anhui Medical University. Patients who had repeated hospitalizations (n = 232) and were absent from urinary tract ultrasonography or CT examination and HBsAg testing (n = 105) were excluded from this study, leaving 1062 patients who constituted the investigated sample. Ethics committee approval was obtained. Since this study was a retrospective and anonymous analysis, no informed consent was needed. We presented the study following the Strengthening the Reporting of Observational studies in Epidemiology (STROBE) guidelines.

According to the presence or absence of UUC, patients were divided into two groups. The diagnosis of UUC was established by urinary tract ultrasonography or computed tomography. The presence of HBsAg was assessed by an enzyme immunoassay. We collected data on some variables that may influence the prevalence of HBV infection, including age, sex, geographical residence, and history of surgery, blood transfusion and haemodialysis. Characteristics of stones and some variables that may affect the formation of urinary calculi were also recorded, including the number of stones; side of stones; staghorn stones; history of UUC; body mass index; hypertension; diabetes mellitus; serum calcium, potassium, uric acid, albumin, alanine aminotransferase, total bilirubin, triglycerides, and creatinine; urinary tract infection, and urinary pH.

### Study outcomes

The primary outcome was the prevalence of HBV infection in individuals with UUC compared with those without UUC. UUC was diagnosed by the presence of one or more stones on ultrasonography or CT examination, where the diameter of the stone was more than 4 mm. The stone composition was confirmed using an infrared spectrometer. Patients were considered chronic HBV infected if they had a history of hepatitis B or had been positive for HBsAg for more than six months. All of the HBsAg-positive patients had not been receiving antiviral therapy six months before and after admission.

The secondary outcome was to investigate the association between the prevalence of HBV infection and the characteristics of stones and clinical and laboratory outcomes in patients with UUC stratified by HBV status. The laboratory outcomes of blood chemistry and urine analysis were obtained after overnight fasting and measured by automated analytical instruments.

### Statistical analysis

Data are shown as the median (first quartile, third quartile), or number (proportion). The categorical variables were determined using the chi-square test (e.g, rates of HBsAg positivity, urinary pH > 6, urinary tract infection, sex, etc.) or Fisher’s exact test (e.g, rates of staghorn stones, history of blood transfusion and haemodialysis), while the means of continuous variables were determined using the Mann–Whitney rank sum test (e.g, urinary pH) or Student’s t test (e.g, age, BMI and other serum biochemical outcomes). To assess risk factors, data were compared using univariate and multivariate logistic regression. The statistical analysis was compared using SPSS® 23.0. *P* < 0.05 was considered statistically significant.

## Results

### Patient characteristics

Among 1062 discharged urological patients, 514 presented with UUC, and 548 did not. The patient characteristics according to the presence or absence of UUC are shown in Table 1 and Additional file 1: Table S1. Compared with patients without UUC, patients with UUC were characterized by male sex and significantly younger age. The geographical residence, and history of surgery, blood transfusion and haemodialysis were not significantly different.

**Table 1:** Characteristics of the 1062 patients stratified by UUC status

	UUC (n = 514)	Non-UUC (n = 548)	*P* value
Age (yr)	52 (43, 62)	62.5 (46, 73)	< 0.001
Sex, n (%)			< 0.001
Males	310 (60.3)	404 (73.7)	
Females	204 (39.7)	144 (26.3)	
Geographical residence, n (%)			0.725
Rural	96 (18.6)	107 (19.5)	
Urban	418 (81.4)	441 (80.5)	
History, n (%)			
Blood transfusion	3 (0.6)	2 (0.4)	0.678
Haemodialysis	3 (0.6)	1 (0.2)	0.359
Surgery	263 (51.2)	254 (46.4)	0.117

UUC, upper urinary calculi. Data are shown as the median (first quartile, third quartile), or number (proportion)

### Overall prevalence of HBsAg-positive individuals

The overall prevalence of HBsAg positivity among the discharged patients in this study was 5.8% (62/1062); it was 6.6% (47/714) in males and 4.3% (15/348) in females, and there was no significant difference (OR 0.639; 95% CI 0.352–1.160; *P* = 0.138) between males and females. The median age of HBsAg-positive patients was 53.5 (44.25, 67) years and that of HBsAg-negative patients was 54 (44, 69) years; the difference was not statistically significant (*P* = 0.666).

### HBsAg positivity in patients with UUC

The prevalence of HBsAg positivity among patients with and without UUC is shown in Table 2. The prevalence of HBsAg positivity (OR 2.175; 95% CI 1.267–3.734; *P* = 0.004) in UUC patients (8.0%) was higher than that in non-UUC patients (3.8%). After stratifying by sex, the relative odds of HBsAg positivity were statistically significant in men but not in women.

**Table 2:** The prevalence of HBsAg positivity in patients with and without UUC

	Crude Prevalence		Unadjusted		Adjusted*	
	UUC (n = 514)	Non-UUC (n = 548)	OR (95% CI)	*P* value	OR (95% CI)	*P* value
Males, n (%)			2.213 (1.205–4.064)	0.009	2.156 (1.162–4.003)	0.015
HBsAg-Positive	29 (9.4)	18 (4.5)				
HBsAg-Negative	281 (90.6)	386 (95.5)				
Females, n (%)			2.938 (0.814–10.604)	0.086	2.947 (0.816–10.643)	0.099
HBsAg-Positive	12 (5.9)	3 (2.1)				
HBsAg-Negative	192 (94.1)	141 (97.9)				

*HBsAg* hepatitis B surface antigen,* UUC* upper urinary calculi,* OR*,odds ratio,* CI* confidence interval, Data are shown as the number (proportion). *Adjusted odds ratios were adjusted for age

**Fig. 1:**
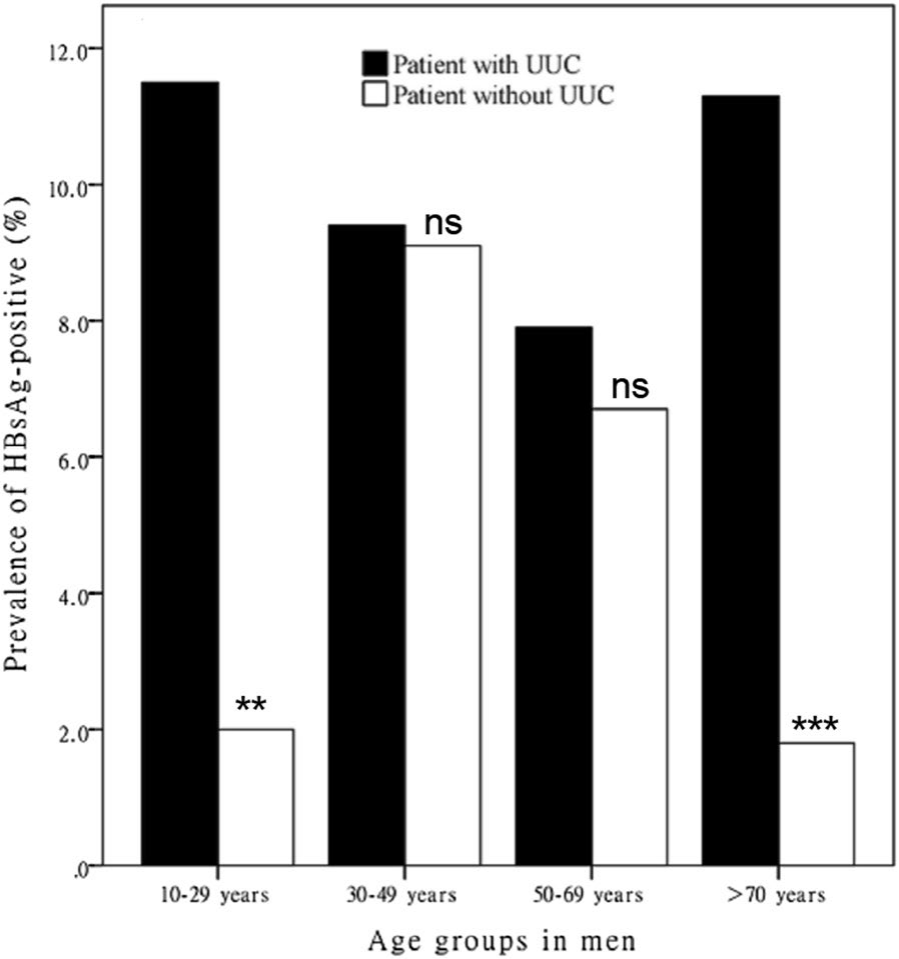
Prevalence of HBsAg positivity among male patients with and without UUC stratified by age. *P < 0.05, **P < 0.01, ***P < 0.001. HBsAg = hepatitis B surface antigen; UUC = upper urinary calculi

The prevalence of HBsAg positivity among male UUC patients was higher than that among male non-UUC patients for all age groups (Fig. 1). Among male UUC patients, a significantly higher prevalence of HBsAg positivity was seen in those groups aged 10 to 29 years and > 70 years.

Potential risk factors that might affect the prevalence of HBV-infected patients were analysed. Age was a strong confounding variable for both men and women. After adjusting for age, the odds of HBsAg positivity remained significantly elevated in male UUC patients compared with male non-UUC patients. However, the adjusted odds of HBsAg positivity in female UUC patients was not significantly higher (Table 2).

The characteristics and laboratory outcomes among UUC patients stratified by HBsAg status are shown in Table 3. HBsAg-positive UUC patients had an elevated urinary pH (mean ranks 327.21 vs 251.46, *P* = 0.001) and a higher percentage of urinary pH > 6 (OR 2.923; 95% CI 1.533–5.575; *P* = 0.001) than HBsAg-negative UUC patients. No significant differences were found for any other variables.

**Table 3:** Characteristics and laboratory outcomes among patients with UUC stratified by HBsAg status

	Patients with UUC		*P* value
	HBsAg-positive (n = 41)	HBsAg-negative (n = 473)	
Age (yr)	50 (43.5, 67)	52 (43, 62)	0.686
BMI (kg/m2)	24.67 (20.99, 26.94)	23.72(21.74, 25.84)	0.284
Sex, n (%)			0.155
Males	29 (70.7)	281 (59.4)	
Females	12 (29.3)	192 (40.6)	
Geographical residence, n (%)			0.127
Rural	37 (90.2)	381 (80.5)	
Urban	4 (9.8)	92 (19.5)	
History, n (%)			
Surgery	16 (39.0)	244 (51.6)	0.123
Blood transfusion	0 (0)	3 (0.6)	1
Haemodialysis	1 (2.4)	2 (0.4)	0.221
Hypertension, n (%)	12 (29.3)	111 (23.5)	0.404
Diabetes mellitus, n (%)	5 (12.2)	61 (12.9)	0.898
UTI, n (%)	13 (31.7)	197 (41.6)	0.214
Urine pH	6.5 (6, 6.5)	6 (5.5, 6.5)	0.001
> 6, n (%)	21 (51.2)	125 (26.4)	0.001
Urine Specific Gravity	1.02 (1.015, 1.025)	1.02 (1.015, 1.025)	0.773
Serum			
Albumin (g/L)	41.4 (40, 42.8)	41.7 (39.1, 44.1)	0.365
Total bilirubin (μmol/L)	14.3 (10.35, 21.1)	15 (11.25, 19.2)	0.519
Direct bilirubin (μmol/L)	3.8 (3.065, 5.62)	3.83 (2.705, 5.29)	0.146
ALT (U/L)	21 (13.5, 34)	20 (13, 30)	0.931
AST (U/L)	21 (17, 26)	19 (15, 24)	0.670
Total cholesterol (mmol/L)	4.66 (4.04, 5.23)	4.69 (4.08, 5.28)	0.635
Triglycerides (mmol/L)	1.1 (0.77, 1.315)	1.17(0.85, 1.49)	0.275
HDL (mmol/L)	1.15 (0.925, 1.285)	1.07 (0.84, 1.305)	0.469
LDL (mmol/L)	2.71 (2.165, 3.28)	2.90 (2.28, 3.46)	0.998
Uric acid (μmol/L)	311 (268.5, 355.5)	337 (278, 389.5)	0.229
Calcium (mmol/L)	2.29 (2.16, 2.355)	2.31 (2.2, 2.4)	0.085
Phosphorus (mmol/L)	1.00 (0.92, 1.105)	1.0284 (0.92, 1.14)	0.636
Sodium (mmol/L)	139 (137.9, 141.45)	139.7(137.7, 141.3)	0.789
Chlorine (mmol/L)	102.4 (100.95, 104.55)	102.4(100.5, 104.5)	0.256
Potassium (mmol/L)	3.97 (3.685, 4.315)	3.93 (3.665, 4.20)	0.592
Creatinine (mmol/L)	83.8 (57.5, 100)	82 (64, 104)	0.835

*HBsAg* hepatitis B surface antigen,* UUC* upper urinary calculi,* BMI*  body mass index,* UTI* urinary tract infection,* AST* aspartate aminotransferase,* ALT* alanine aminotransferase,* LDL* low-density lipoprotein,* HDL* high-density lipoprotein. Data are shown as the median (first quartile, third quartile), or number (proportion)

### HBsAg positivity and characteristics of stones in UUC individuals

The stone characteristics among patients with UUC stratified by HBsAg status are shown in Table 4. HBsAg-positive UUC patients had a higher percentage of staghorn stones (OR 2.624; 95% CI 1.136–6.064; *P* = 0.043) than HBsAg-negative UUC patients. The number of stones, side of stones, and history of UUC were not significantly different.

**Table 4:** Characteristics of stones among patients with UUC stratified by HBsAg status

	Patients with UUC		OR (95% CI)	*P* value
	HBsAg-positive (n = 41)	HBsAg-negative (n = 473)		
Number of stones, n (%)			1.029 (0.541–1.957)	0.930
1	18 (43.9)	211 (44.6)		
≥ 2	23 (56.1)	262 (55.4)		
Side of stones, n (%)			1.267 (0.652–2.462)	0.739
Unilateral	26 (63.4)	325 (68.7)		
Bilateral	15 (36.6)	148 (31.3)		
Staghorn stone, n (%)	8 (19.5)	40 (8.5)	2.624 (1.136- 6.064)	0.043
History of UUC, n (%)	12 (29.2)	132 (27.9)	1.069 (0.530–2.157)	0.852

HBsAg, hepatitis B surface antigen; UUC, upper urinary calculi; OR, odds ratio; CI, confidence interval. Data are shown as the number (proportion)

## Discussion

The formation of urinary calculi is a complex multifactor process that includes internal factors (such as sex, age and genetics) and external factors (such as climate, geography, diet, water intake and mineral composition etc.) [10]. The potential risk factors for urinary calculi include metabolic disorders, hypercalcemic disorders, low urine volume, recurrent urinary tract infections, hypertension, obesity, abnormalities of urinary pH, repeated urinary tract infections, and renal tubular acidosis [11–13]. However, some of these risk factors are not always associated with urinary calculi and may vary greatly between men and women.

HBV infection has been associated with various kidney diseases and commonly presents with arterial hypertension, membranous nephropathy, chronic kidney disease and renal tubular acidosis [8, 9, 14, 15], which may be affected by immune complex deposition or direct glomerular and tubular injury and antiviral therapy^.^ [15, 16] Recently, Guimerà et al*.* [17] showed that renal tubular acidosis has been confirmed as a pathogenic factor for urinary calculi, and although calcium oxalate stones are the dominant type of urinary calculi among the general population, calcium phosphate stones are most common in individuals with renal tubular acidosis. A retrospective study showed that there was a high prevalence of urolithiasis among patients with thin basement membrane nephropathy, mainly in the presence of hypercalciuria and hyperuricosuria [18]. Several recent studies have shown that the early stage of chronic kidney disease is usually associated with hypocitraturia, and insignificant metabolic acidosis also causes hypocitraturia to increase urinary pH [19–21].

Despite numerous studies on the association between kidney diseases and urinary calculi [22], whether HBV infection is related to an elevated risk of urinary calculi remains unclear. In this work involving discharged urological patients after stratifying by sex, we found that chronic HBV infection was strongly associated with UUC, at least in men. The cause for the associated sex-specific differences between UUC and HBV infection is unclear. The risk factors and pathophysiology of UUC formation in men may be different from those in women. Otherwise, we may have failed to find a significant difference between HBV infection and UUC in women due to the small number of female patients in our study.

The present investigation demonstrated that urine pH was the only independent predictor for UUC formation in HBsAg-positive patients. Among UUC patients, the prevalence of urinary pH > 6 in HbsAg-positive patients was significantly higher than that in HbsAg-negative patients. The urinary pH > 6 often indicates a diagnosis of suspected renal tubular acidosis, although an acidification test is needed. Several other studies [17, 23, 24] have also shown that the prevalence of kidney stones was significantly higher in individuals with high urinary pH levels than in those with normal urinary pH and tended to be correlated with calcium phosphate stones, which easily recur and form staghorn stones [25]. These findings may explain why the incidence of staghorn stones in HbsAg-positive UUC patients in this study was higher than that in HbsAg-negative UUC patients.

The mechanisms by which chronic HBV infection promotes the occurrence and development of UUC, especially in patients with high levels of urine pH, are not known. The possible mechanisms may include an imbalance between the kidneys’ ability to excrete H^+^ ions and resorb HCO_3_^−^ resulting in elevated urinary pH, which can in turn lead to increased calcium phosphate crystal precipitation [26]. Increased urinary pH might be a potential link between UUC and chronic HBV infection by increasing phosphate saturation in the urine. Presumably, in patients with chronic HBV infection, UUC may be mainly the calcium phosphate type, but to date, no studies have evaluated this aspect.

Direct infection of the renal tubular epithelium by HBV may play a role in the occurrence and development of UUC. Diao et al*. *[27] proved that HBV can directly infect renal tubular epithelial cells and that the replication of HBV increases the apoptosis of renal tubular cells. In vitro research has demonstrated that by upregulating Fas gene expression, serum from patients infected with hepatitis B virus promotes apoptosis of human proximal renal tubular epithelial cells^.^ [28] Apoptosis and injury of renal tubular epithelial cells were able to induce subsequent stone formation [29]. Some authors have reported that damaged cells develop lesions that induce the retention of particles on the surface of renal papillae, and crystals become attached to damaged renal tubular cells [30, 31]. In addition, both UCC and HBV infection may share a common pathogenesis or exacerbating factors as Porter et al. [32] reported and immunosuppression could promote both HBV infection and urinary tract infections, while pathogens such as* E. coli* in urine could promote the formation of stones [33]. HBV infection of renal tubular epithelial cells may increase the risk of UUC formation by causing changes in renal tubular acid function or promoting the accumulation and growth of calcium crystals. Further research is needed to verify this interesting hypothesis.

The major strength of our study is that the association between chronic HBV infection and UUC was confirmed. To our knowledge, our work fills a gap in the understanding of the relationship between these two high-incidence diseases. Another strength is that we pointed out the potential risk factors for formatting urinary calculi to chronic HBV infection patients and provided important evidence for further research.

However, some limitations should be considered. This study was a retrospective study and some data were absent, such as 24-h urinary Ca and P and HBV load in urine and kidney biopsies, making it difficult to determine the mechanism of how hepatitis B plays a role in the formation of kidney stones. Further prospective clinical studies and basic experiments are hopeful to determine the potential mechanism. Another potential limitation is that because the information related to HBV in the normal population is private and almost inaccessible, the control group included patients who needed hospitalization due to urological disease. However, these diseases will not affect the prevalence of HBV infection in these hospitalized patients, so we believe that the results of the comparison are reliable.

## Conclusions

This study showed that chronic HBV infection was strongly associated with UUC. The urinary pH > 6 and staghorn stones were more common in UUC patients with chronic HBV infection. To our knowledge, these findings are the first to be reported. Further studies are necessary to determine the potential mechanism between HCV infection and the formation of UUC.

## Supplementary Information

**Additional file 1:** Kinds of diseases in 1062 patients.

## Data Availability

The datasets supporting the conclusions of this article are included within the article and its additional file.

## Abbreviations

HBV: Hepatitis B virus
UUC: Upper urinary calculi
HBsAg: Hepatitis B surface antigen
