# Supplementary material for: Chronic hepatitis B virus infection increases the risk of upper urinary calculi
Source: BMC Urol. 2022 Jun 6;22:82. doi: 10.1186/s12894-022-01038-z (PMC9169271; doi:10.1186/s12894-022-01038-z)
Supplement: Supplementary file 1 — Additional file 1: Kinds of diseases in 1062 patients. [file 12894_2022_1038_MOESM1_ESM.doc]

Supplementary Table 1. Kinds of diseases in 1062 patients

| UUC (n = 514) | Non-UUC (n = 548) |
| --- | --- |
| Kidney stones，309（60.1%） | Benign prostatic hyperplasia, 126 (23%) |
| Ureteral stones, 205 (39.9%) | Infectious diseases, 43 (7.8%) |
|  | Malignant tumor, 88 (16.1%) |
|  | Benign tumor, 76 (13.9%) |
|  | Varicocele, 42 (7.7%) |
|  | Neurogenic bladder disease, 36 (6.6%) |
|  | Traumatic disease, 29 (5.3%) |
|  | Congenital diseases, 67 (12.2%) |
|  | renal cyst, 21 (3.8%)  Stress incontinence, 9 (1.6%)  [testicular](javascript:;) [torsion](javascript:;), 5 (0.9%)  [urinary](javascript:;) [fistula](javascript:;), 3 (0.5%)  undefined, 3 (0.5%) |

NOTE. UUC = upper urinary calculi. Data are shown as number (proportion).
